# The complete chloroplast DNA sequence of the green alga *Oltmannsiellopsis viridis *reveals a distinctive quadripartite architecture in the chloroplast genome of early diverging ulvophytes

**DOI:** 10.1186/1741-7007-4-3

**Published:** 2006-02-03

**Authors:** Jean-François Pombert, Claude Lemieux, Monique Turmel

**Affiliations:** 1 Département de biochimie et de microbiologie, Université Laval, Québec, Canada

## Abstract

**Background:**

The phylum Chlorophyta contains the majority of the green algae and is divided into four classes. The basal position of the Prasinophyceae has been well documented, but the divergence order of the Ulvophyceae, Trebouxiophyceae and Chlorophyceae is currently debated. The four complete chloroplast DNA (cpDNA) sequences presently available for representatives of these classes have revealed extensive variability in overall structure, gene content, intron composition and gene order. The chloroplast genome of *Pseudendoclonium *(Ulvophyceae), in particular, is characterized by an atypical quadripartite architecture that deviates from the ancestral type by a large inverted repeat (IR) featuring an inverted rRNA operon and a small single-copy (SSC) region containing 14 genes normally found in the large single-copy (LSC) region. To gain insights into the nature of the events that led to the reorganization of the chloroplast genome in the Ulvophyceae, we have determined the complete cpDNA sequence of *Oltmannsiellopsis viridis*, a representative of a distinct, early diverging lineage.

**Results:**

The 151,933 bp IR-containing genome of *Oltmannsiellopsis *differs considerably from *Pseudendoclonium *and other chlorophyte cpDNAs in intron content and gene order, but shares close similarities with its ulvophyte homologue at the levels of quadripartite architecture, gene content and gene density. *Oltmannsiellopsis *cpDNA encodes 105 genes, contains five group I introns, and features many short dispersed repeats. As in *Pseudendoclonium *cpDNA, the rRNA genes in the IR are transcribed toward the single copy region featuring the genes typically found in the ancestral LSC region, and the opposite single copy region harbours genes characteristic of both the ancestral SSC and LSC regions. The 52 genes that were transferred from the ancestral LSC to SSC region include 12 of those observed in *Pseudendoclonium *cpDNA. Surprisingly, the overall gene organization of *Oltmannsiellopsis *cpDNA more closely resembles that of *Chlorella *(Trebouxiophyceae) cpDNA.

**Conclusion:**

The chloroplast genome of the last common ancestor of *Oltmannsiellopsis *and *Pseudendoclonium *contained a minimum of 108 genes, carried only a few group I introns, and featured a distinctive quadripartite architecture. Numerous changes were experienced by the chloroplast genome in the lineages leading to *Oltmannsiellopsis *and *Pseudendoclonium*. Our comparative analyses of chlorophyte cpDNAs support the notion that the Ulvophyceae is sister to the Chlorophyceae.

## Background

The green algae are divided into the phyla Streptophyta and Chlorophyta. The Streptophyta (sensu Bremer [1]) encompasses the algae from the class Charophyceae and all land plants, whereas the Chlorophyta (sensu Sluiman [2]) contains algae from the classes Prasinophyceae, Ulvophyceae, Trebouxiophyceae and Chlorophyceae [3]. The basal position of the Prasinophyceae in the Chlorophyta is generally well established, but the branching order of the Ulvophyceae, Trebouxiophyceae and Chlorophyceae (UTC) remains a matter of debate [4-6]. It has been proposed that a third lineage at the base of the Streptophyta and Chlorophyta is represented by *Mesostigma viride *[7-9], an alga traditionally classified within the prasinophytes. This green plant lineage, however, is debated, as some studies suggest that *Mesostigma *is an early offshoot of the phylum Streptophyta [10-12].

Investigations of chloroplast DNA (cpDNA) from green algae representing each of the five recognized classes have revealed that the genomes of the charophyte *Chaetosphaeridium globosum *[13] and the prasinophytes *Mesostigma *[7] and *Nephroselmis olivacea *[14] are highly similar to those of land plants. Like most land plants cpDNAs, these green algal genomes are partitioned into a quadripartite architecture by two copies of a large inverted repeat (IR) separating small (SSC) and large (LSC) single copy regions. Most notably, the great majority of the genes occupying a given single copy region in prasinophyte genomes map to the same single copy region in *Chaetosphaeridium *and land plant cpDNAs. The increased structural stability of the chloroplast genome conferred by the IR sequence has been hypothesized to limit gene exchanges between the SSC and LSC regions [15]. The IR region readily expands or contracts and thus can easily gain or lose genes from the neighbouring single copy regions through a process known as the ebb and flow [16]. Despite its variable gene content, the IR always features the ribosomal RNA (rRNA) operon (*rrs*-*I*(gau)-*A*(ugc)-*rrl*-*rrf*) and this operon is always transcribed toward the SSC region. In addition to their characteristic pattern of gene partitioning, prasinophyte and streptophyte chloroplast genomes share a number of features that were most probably inherited from the progenitor of all green plant cpDNAs. First, they have retained several gene clusters that date back to the cyanobacterial ancestor of all chloroplasts. Second, their genes are densely packed and their intergenic regions virtually lack short dispersed repeats (SDRs). Finally, with 128 to 137 genes, their gene repertoire is one of the largest among green plant cpDNAs.

In contrast, the chloroplast genome has been substantially reorganized in the UTC. The quadripartite architecture has been lost from the genome of the trebouxiophyte *Chlorella vulgaris *[17] following the disappearance of one copy of the IR sequence. Although the quadripartite architecture has been retained in the genome of the ulvophyte *Pseudendoclonium akinetum *[6], the IR sequence is atypical in featuring a rRNA operon transcribed towards the LSC region [6]. In addition, the pattern of gene partitioning within the SSC/LSC regions of *Pseudendoclonium *cpDNA deviates significantly from those found in its prasinophyte and land plant counterparts; the small single copy region of this ulvophyte genome includes 14 genes that are usually located within the LSC region. In the chlorophycean alga *Chlamydomonas reinhardtii *[18], the two single copy regions are similar in size and the genes are so thoroughly scrambled that no distinction is possible between the SSC and LSC regions. The *Chlorella, Pseudendoclonium *and *Chlamydomonas *chloroplast genomes have lost many of the ancestral gene clusters that are shared between *Mesostigma *and *Nephroselmis *cpDNAs, feature a reduced gene content (from 94 genes in *Chlamydomonas *to 112 genes in *Chlorella*) compared to prasinophyte and streptophyte genomes, and contain SDRs in their intergenic regions. The low density of coding sequences in these genomes is explained not only by the smaller number of genes but also by the expansion of intergenic regions. Moreover, unlike *Mesostigma *and *Nephroselmis *cpDNAs, the chloroplast genomes of the three UTC algae have acquired group I introns (from three in *Chlorella *to 27 in *Pseudendoclonium*) and group II introns (two in *Chlamydomonas*).

To gain insights into the nature of the events that led to the reorganization of the chloroplast genome in the Ulvophyceae, we have determined the complete cpDNA sequence of *Oltmannsiellopsis viridis*. This marine unicellular green alga exhibits a counterclockwise arrangement of basal bodies [19,20] and a single cup-shaped chloroplast [20]. Previously classified in the Chlorophyceae [19,21], *Oltmannsiellopsis *is currently considered to be the type species of the order Oltmannsiellopsidales (Ulvophyceae) [22]. The Oltmannsiellopsidales have been shown to branch at the base of the Ulvophyceae [4] and have been used as outgroup for phylogenetic analyses of the Ulvophyceae [23-25]. Considering that *Pseudendoclonium *represents a distinct, early diverging lineage of the Ulvophyceae (Ulotrichales, see supplementary Figure S1 in [6]), identification of the set of features common to *Oltmannsiellopsis *and *Pseudendoclonium *cpDNAs should throw light into the chloroplast genome architecture of the earliest diverging ulvophytes and, accordingly, into the cpDNA changes that occurred in the separate lineages leading to *Oltmannsiellopsis *and *Pseudendoclonium*. We found that the IR-containing genome of *Oltmannsiellopsis *differs considerably from its *Pseudendoclonium *and other chlorophyte counterparts in intron content and gene order, but shares closer similarities with *Pseudendoclonium *cpDNA in terms of quadripartite architecture, gene content and gene density. In the context of the debate concerning the branching order of the UTC lineages, the predicted architecture of the chloroplast genome of the earliest members of the Ulvophyceae strengthens the notion that this lineage is sister to the Chlorophyceae [5,6].

## Results and discussion

### General features

**Figure 1:**
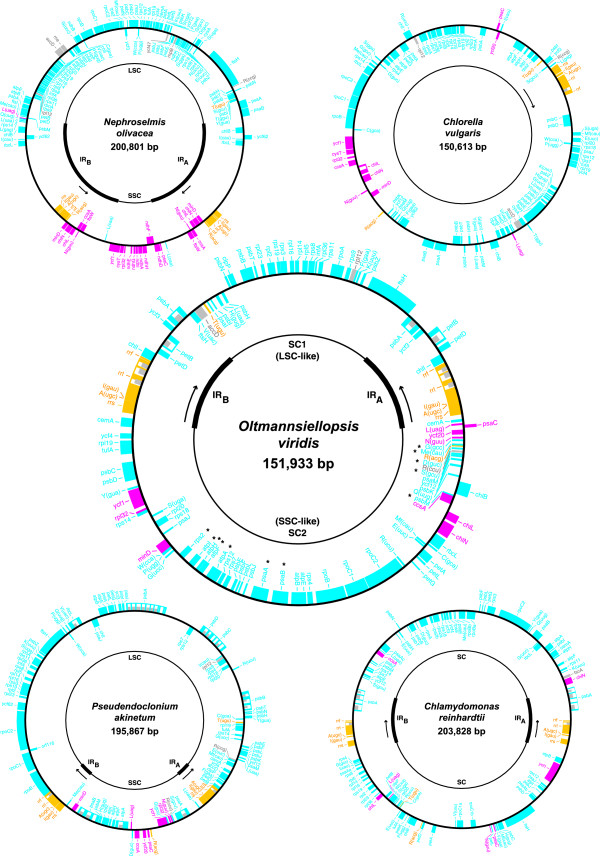
**Gene maps of *Oltmannsiellopsis *and other chlorophyte cpDNAs**. Genes (filled boxes) outside each map are transcribed clockwise. The transcription direction of the rRNA genes is indicated by arrows. Genes shown in yellow, blue and red map to the IR, LSC and SSC regions in *Mesostigma *cpDNA, respectively. On the *Oltmannsiellopsis *map, genes characteristic of the LSC region that reside in the single copy region corresponding to SSC in both *Oltmannsiellopsis *and *Pseudendoclonium *cpDNAs are denoted by asterisks. Genes absent from *Mesostigma *cpDNA are shown in grey. tRNA genes are indicated by the one-letter amino acid code followed by the anticodon in parentheses (Me, elongator methionine; Mf, initiator methionine). A total of five introns (open boxes) were identified, some of which feature ORFs (narrow boxes).

Table 1 compares the general features of *Oltmannsiellopsis *cpDNA [GenBank: DQ291132] with those of the four chlorophyte cpDNAs completely sequenced thus far, *i.e*. the genomes of *Nephroselmis *[GenBank:NC_000927], *Chlorella *[GenBank:NC_001865], *Pseudendoclonium *[GenBank:AY835431] and *Chlamydomonas *[GenBank:NC_005353]. At 59.5%, the overall A+T content of *Oltmannsiellopsis *cpDNA is similar to that of *Nephroselmis *cpDNA but is significantly lower than those of the three previously sequenced UTC genomes. The *Oltmannsiellopsis *genome maps as a circular molecule of 151,933 bp (Figure 1) and contains 105 genes. Two copies of an IR sequence of 18,510 bp, each encoding ten genes, are separated from one another by unequal single copy regions, designated SC1 and SC2. Like other UTC cpDNAs, the *Oltmannsiellopsis *genome is less densely packed with coding sequences than *Mesostigma *and *Nephroselmis *cpDNAs; at 59.2%, its density of coding sequences is similar to those of *Chlorella *and *Pseudendoclonium *cpDNAs. Intergenic spacers in *Oltmannsiellopsis *cpDNA feature SDRs and have an average size of 512 bp, a value comparable to that observed for *Pseudendoclonium *cpDNA (600 bp). A total of five introns, all of which belong to the group I family, were identified in *Oltmannsiellopsis *cpDNA.

### Gene and intron contents

The gene content of *Oltmannsiellopsis *cpDNA is intermediate between those of *Chlorella *and *Chlamydomonas *cpDNAs (Table 1). Although *Oltmannsiellopsis *and *Pseudendoclonium *cpDNAs encode the same number of genes, these genomes differ slightly in their gene repertoire (Table 2). *Oltmannsiellopsis *cpDNA has retained all three *chl *genes that are missing from *Pseudendoclonium *cpDNA but has lost *ycf62*, *trnL*(caa) and *trnR*(ccg). Relative to *Chlorella *cpDNA, the genomes of *Oltmannsiellopsis*, *Pseudendoclonium *and *Chlamydomonas *are missing a set of five genes, *i.e. cysA*, *cyst*, and three tRNA genes (*trnL*(gag), *trnS*(gga) and *trnT*(ggu)) (Table 2). The absence of three genes (*ycf62*, *trnL*(caa) and *trnR*(ccg)) is uniquely shared by *Oltmannsiellopsis *and *Chlamydomonas *cpDNAs, whereas no specific gene loss is shared by *Pseudendoclonium *and *Chlamydomonas *cpDNAs. Both *Oltmannsiellopsis *and *Pseudendoclonium *cpDNAs have retained the *trnR*(ccu) gene, which is absent from all other completely sequenced chlorophyte cpDNAs.

As in the UTC chloroplast genomes previously investigated, the coding regions of several genes in *Oltmannsiellopsis *cpDNA are expanded relative to their *Mesostigma *counterparts [6] (Table 3). However, most of the gene expansions in *Oltmannsiellopsis *are less extensive than those in *Pseudendoclonium*; only *cemA *displays a longer coding sequence than its *Pseudendoclonium *homologue.

Our finding of five group I introns in *Oltmannsiellopsis *cpDNA contrasts sharply with the 27 group I introns found in *Pseudendoclonium *cpDNA [6] (Table 1). The lower abundance of introns in *Oltmannsiellopsis *cpDNA mainly accounts for the smaller size of this genome relative to *Pseudendoclonium* cpDNA. The *Oltmannsiellopsis *introns interrupt three genes (*petB*, *psbA*, and *rrl*) found in the IR (Table 4). The *petB *and *psbA *genes each contain one intron, whereas three introns are present in *rrl*. All five introns, with the exception of the *petB *intron, are positionally and structurally homologous to previously reported introns in green plant cpDNAs (Table 5). While homologues of the *Oltmannsiellopsis psbA *intron are present in *Pseudendoclonium *and *Chlamydomonas*, homologues of the three *rrl *introns are found in a larger diversity of green plants. Considering that these homologous introns have been identified in UTC lineages, they could have been inherited by vertical inheritance from the last common ancestor of UTC algae; however, the finding that they potentially code for homing endonucleases of the LAGLIDADG or GIY-YIG families (Table 4) does not allow us to exclude the possibility that they were acquired by horizontal transfer. Although most of the 16 group I introns in *Pseudendoclonium *cpDNA have no homologues at identical cognate sites in other chloroplast genomes, their close structural and sequence similarities together with their absence from *Oltmannsiellopsis *cpDNA suggest that they arose from intragenomic proliferation in the lineage leading to *Pseudendoclonium *[6]. Note that Blast searches of the *Oltmannsiellopsis petB *intron sequence against the GenBank database failed to detect any homologous intron in other organisms.

### Genome structure and gene partitioning

The pattern of gene partitioning within the single copy regions of *Oltmannsiellopsis *cpDNA differs substantially from the ancestral partitioning pattern observed for *Mesostigma*, *Nephroselmis *and streptophyte cpDNAs (Figure 1). The great majority of the 30 genes found in the SC1 region of *Oltmannsiellopsis *are typically found in the ancestral LSC region, whereas the SC2 region contains 52 genes characteristic of the ancestral LSC region in addition to ten genes characteristic of the ancestral SSC region. Interestingly, SC2 includes 12 of the 14 LSC genes that have been transferred to the SSC region in *Pseudendoclonium *cpDNA. The two exceptional *Pseudendoclonium *genes that have no homologues in *Oltmannsiellopsis *SC2 are *trnH*(gug) and *trnL*(caa); the *trnH*(gug) gene resides in the SC1 region of *Oltmannsiellopsis*, whereas *trnL*(caa) has been lost from *Oltmannsiellopsis *cpDNA. Considering the gene contents of the *Oltmannsiellopsis *single copy regions, it appears inappropriate to label these regions according to their sizes. Although SC1 is smaller than SC2, it likely corresponds to the ancestral LSC region, and SC2 is apparently derived from the ancestral SSC region.

The IR sequence in *Oltmannsiellopsis *cpDNA is about 12 kb larger than that in *Pseudendoclonium *cpDNA and contains five genes in addition to those found in the rRNA operon (Figure 1). At 18,510 bp, the IR sequence of *Oltmannsiellopsis *is similar in size to that of *Chlamydomonas *(Table 1). Both IR junctions in *Oltmannsiellopsis *cpDNA encompass genes (*cemA *and *ftsH*) of which the coding sequences expand into the single copy regions. As in the *Pseudendoclonium *IR, the *Oltmannsiellopsis *rRNA genes are transcribed towards the single copy region carrying the genes that map to the LSC in prasinophyte and streptophyte cpDNAs. In contrast, the rRNA operon is transcribed toward the SSC region in *Nephroselmis *and streptophyte cpDNAs. The orientation of the rRNA operon cannot be established in *Chlamydomonas *cpDNA owing to the extensively scrambled single copy regions, and this orientation remains unknown in *Chlorella *cpDNA because of the IR loss.

Considering that *Oltmannsiellopsis *and *Pseudendoclonium *represent distinct, early diverging lineages of the Ulvophyceae, the striking similarities between the quadripartite architectures of *Oltmannsiellopsis *and *Pseudendoclonium *cpDNAs suggest that both the atypical gene partitioning pattern and unusual orientation of the IR were characteristic of the chloroplast genome of earliest-diverging ulvophytes. Our data predict that the SSC region of the last common ancestor of *Oltmannsiellopsis *and *Pseudendoclonium *cpDNAs featured 12 of the genes usually found in the LSC region in *Nephroselmis *and streptophyte cpDNAs, whereas the LSC region contained exclusively genes characteristic of the ancestral LSC region. Consequently, in the lineage leading to *Pseudendoclonium*, two extra genes were transferred to the SSC region, whereas 40 additional genes migrated to this region in the *Oltmannsiellopsis *lineage. Although the mechanisms underlying these gene migrations between single copy regions remain unknown, they probably involved intramolecular or intermolecular recombination events. The analysis of conserved gene clusters reported below clearly indicates that several genes were transferred together in the course of these migrations.

Genes have been more extensively shuffled between the two single copy regions in *Chlamydomonas *cpDNA (Figure 1). It can be envisioned that during the evolution of ulvophytes and chlorophycean green algae, the ancestral pattern of gene partitioning was disrupted in successive steps, with a *Pseudendoclonium*-like organization evolving into an *Oltmannsiellopsis*-like organization, leading ultimately to the extensive scrambling of genes observed in *Chlamydomonas*. Given the absence of the IR from the *Chlorella *genome, it is very difficult to ascertain whether the transcription direction of the rRNA operon changed and whether genes were relocated from one genomic region to another during the evolution of trebouxiophytes. Loss of the IR is usually associated with many gene rearrangements [15]; in the case of *Chlorella *cpDNA, however, all the genes usually found in the ancestral SSC region have remained clustered, with the exception of three genes (*psaC*, *ycf20 *and *trnL*(uag)) (Figure 1). Investigations of IR-containing chloroplast genomes from distinct trebouxiophyte lineages will be required to test whether some of the gene relocations identified here in both *Oltmannsiellopsis *and *Pseudendoclonium *cpDNAs originated from the common ancestor of UTC algae.

### Gene clustering

**Figure 2:**
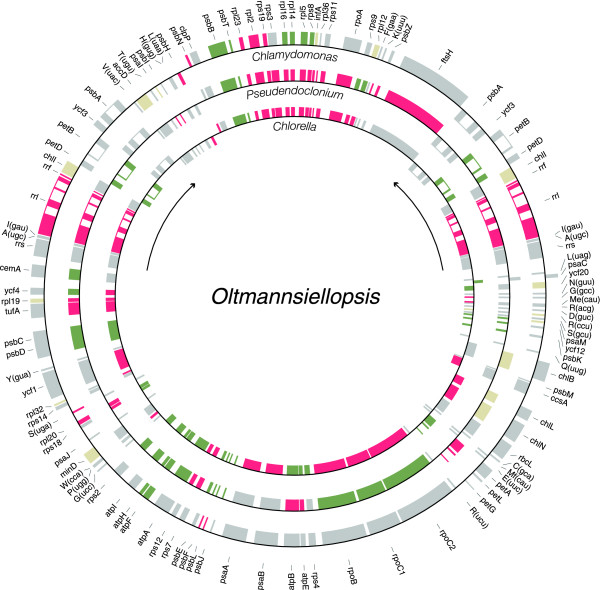
**Gene clusters shared between *Oltmannsiellopsis *and other UTC algal cpDNAs**. Shared clusters are shown on the *Oltmannsiellopsis *gene map as alternating series of green and red boxes. Genes located outside conserved clusters are shown in grey. Genes missing from *Chlamydomonas*, *Pseudendoclonium *and *Chlorella *are represented in beige. The extent of the IR and transcription direction of the rRNA genes are denoted by arrows. Genes outside the map are transcribed clockwise.

The overall gene organization of *Oltmannsiellopsis *cpDNA differs extensively from that of its *Pseudendoclonium *homologue and, surprisingly, more closely resembles that of *Chlorella *cpDNA (Figure 2). *Oltmannsiellopsis *and *Chlorella *cpDNAs share 21 blocks of colinear sequences that contain a total of 65 genes, whereas *Oltmannsiellopsis *and *Pseudendoclonium *cpDNAs have in common 18 blocks containing 55 genes. Only eight blocks containing 19 genes are conserved in the *Oltmannsiellopsis *and *Chlamydomonas *genomes.

**Figure 3:**
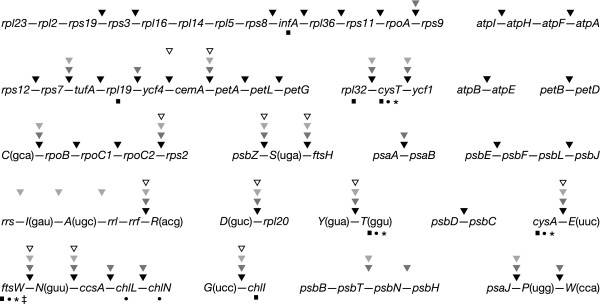
**Fragmented ancestral gene clusters in the cpDNAs of UTC algae**. The indicated clusters are found in both *Mesostigma *and *Nephroselmis *cpDNAs. Note that *rpl22 *has not been represented in the large ribosomal protein cluster because it is present only in *Mesostigma *cpDNA (between *rps19 *and *rps3*). Sites of fragmentation are denoted by arrowheads of different shades above the clusters: *Chlamydomonas*, filled arrowheads; *Pseudendoclonium*, dark grey arrowheads; *Oltmannsiellopsis*, light grey arrowheads; and *Chlorella*, open arrowheads. Genes missing from *Chlamydomonas*, *Pseudendoclonium*, *Oltmannsiellopsis *and *Chlorella *are indicated by squares, circles, asterisks and double dagger, respectively. Gene polarities are not shown.

Many of the 24 ancestral gene clusters shared by *Mesostigma *and *Nephroselmis *cpDNAs have been disrupted during the evolution of the UTC green algae. In this study, we have analyzed 19 ancestral clusters; the five remaining ones could not be investigated because the genes they contain have been lost from UTC cpDNAs (Figure 3). All 19 clusters have been broken at least in one occasion during the evolution of the UTC algae. With only 12 breakpoints, *Chlorella *cpDNA displays the strongest conservation of ancestral clusters. With 20 breakpoints, *Oltmannsiellopsis *cpDNA occupies a median position between *Chlorella *and *Pseudendoclonium *(24 breakpoints) cpDNAs, whereas *Chlamydomonas *cpDNA reveals twice as many breakpoints (42 breakpoints). The *Chlamydomonas, Oltmannsiellopsis *and *Pseudendoclonium *genomes share five breakpoints that are missing in *Chlorella *cpDNA. Aside from these breakpoints, *Pseudendoclonium *and *Chlamydomonas *cpDNAs share six breakpoints that are absent from *Oltmannsiellopsis *and *Chlorella *cpDNAs. There is no breakpoint exclusive to the *Oltmannsiellopsis *and *Chlamydomonas *genomes.

Two ancestral clusters display breakpoints that are unique to the Ulvophyceae. The almost universally conserved *psbB*-*psbT*-*psbN*-*psbH *cluster was fragmented at the 5' end of *psbN*, creating two separate pieces, each encoding a pair of genes, in *Oltmannsiellopsis *cpDNA. In the *Pseudendoclonium *lineage, the introduction of an additional breakpoint on the opposite side of *psbN *led to the relocation of this gene on the DNA strand encoding *psbB*, *psbT *and *psbH*, without any change in gene order. In the *Oltmannsiellopsis *lineage, three breakpoints occurred in the ancestral rRNA operon to generate a new transcription unit in which the order of the *trnA*(ugc) and *trnI*(gau) genes has been reversed. Rearranged rRNA operons have been reported for the cpDNAs of the trebouxiophyte *Chlorella ellipsoidea *[26] and the ulvophyte *Codium fragile *[27]; however, in these cases, the ancestral rRNA operon was split into separate fragments that are transcribed from different promoters.

**Figure 4:**
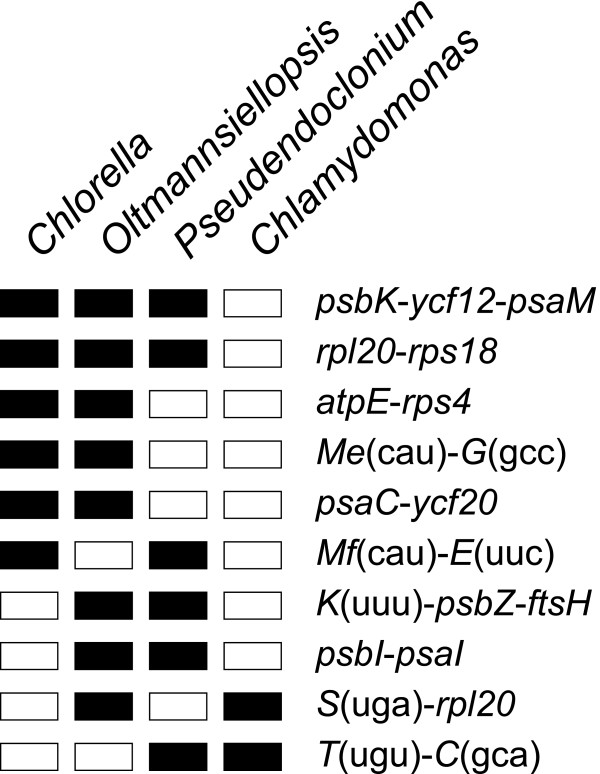
**Derived gene clusters shared between the cpDNAs of UTC algae**. Filled/open boxes represent the presence/absence of clusters. Gene polarities are not shown.

In terms of derived gene clusters, *Oltmannsiellopsis *cpDNA is most similar to *Chlorella *cpDNA (Figure 4). A derived cluster is defined here as a group of genes with the same relative polarities in two or more UTC genomes, but absent from *Mesostigma *and *Nephroselmis *cpDNAs. *Oltmannsiellopsis *cpDNA shares five derived clusters with its *Chlorella *homologue, whereas *Pseudendoclonium *cpDNA shares three clusters, one of which is missing from *Oltmannsiellopsis*. Of the four derived clusters common to *Oltmannsiellopsis *and *Pseudendoclonium *cpDNAs, none is found in *Chlamydomonas *cpDNA.

We estimated that a minimum of 50 inversions would be required to transform the gene organization of *Oltmannsiellopsis *cpDNA into that of any other chlorophyte genome (Table 6). Comparative analyses of cpDNAs from land plants [15] and from closely related chlamydomonads [28,29] suggest that inversions represent the predominant mechanism of chloroplast genome rearrangements in green plants. However, inversions might be not the only mutational events causing gene order changes in chlorophytes cpDNAs, as transpositions have been proposed to account for some of the rearrangements observed in Campanulaceae [30] and in subclover [31] cpDNAs.

### Repeated elements

**Figure 5:**
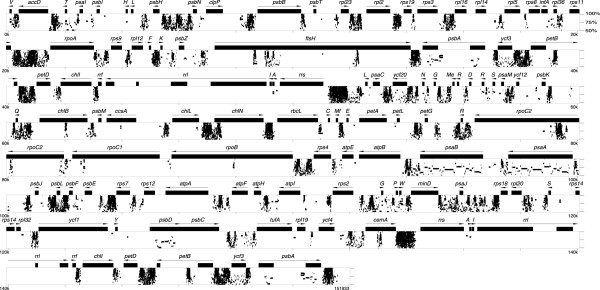
**Positions of SDR elements in *Oltmannsiellopsis *cpDNA**. The *Oltmannsiellopsis *cpDNA sequence was aligned against itself using PipMaker. Regions containing SDRs can be identified as clusters of dots. Similarities between aligned regions are shown as average percent identity (between 50 and 100% identity). Genes and their polarities are denoted by horizontal arrows and coding sequences are represented by filled boxes.

**Figure 6:**
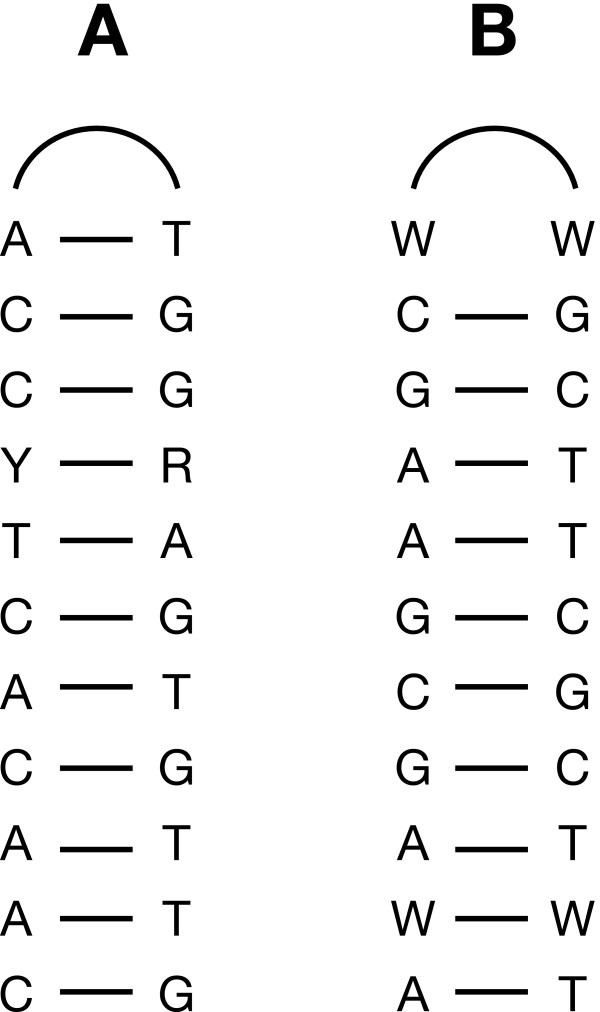
Predicted secondary structures formed by SDR units A and B in *Oltmannsiellopsis *cpDNA.

A large number of SDR elements are found in *Oltmannsiellopsis *cpDNA (Figure 5). Although these elements reside predominantly within intergenic spacers and introns, a few copies populate the coding regions of *cemA*, *chlB*, *chlL*, *chlN*, *ftsH*, *rpoB*, *rpoC1 *and *rpoC2*. The most abundant elements can be classified into five groups of non-overlapping repeat units (A through E) on the basis of their primary sequences (Table 7). Their sizes range from 7–21 bp and their copy numbers vary from 17 to more than 250. The sequence of repeat unit A or B is most often linked to the reverse complement of the same sequence, thus forming perfect palindromes or putative stem-loop structures with a loop of two A or two T (Figure 6). In some instances, the palindromes or stem portions of the stem-loop structures are extended by the addition of less frequent repeats. Furthermore, a few copies of repeat units A and B occur as solitary sequences, representing probably degenerated versions of the more common arrangements featuring palindromes or stem-loop structures. Repeat unit C can form stem-loop structures, with a loop of variable size. Although repeat units D and E are not associated with stem-loop structures, they reside in the vicinity of other repeated elements.

The SDRs in *Oltmannsiellopsis *cpDNA do not closely resemble those present in other UTC cpDNAs. The *Oltmannsiellopsis *repeats are biased in G+C, whereas the *Chlorella *repeats show a bias in A+T. The *Pseudendoclonium *and *Chlamydomonas *SDRs are also rich in G+C, but their sequences share no obvious similarities with the *Oltmannsiellopsis *repeats. This lack of sequence similarities between SDRs derived from distinct UTC genomes suggests that SDRs have been acquired independently in UTC lineages. However, the alternative hypothesis that SDRs were transmitted vertically cannot be excluded if we assume that these elements evolve at a very fast pace. Studies of cpDNAs from closely related UTC taxa will be required to distinguish between these two hypotheses.

**Figure 7:**
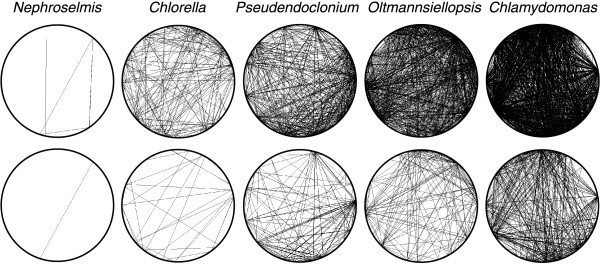
**Densities of SDR elements in *Oltmannsiellopsis *and other chlorophyte cpDNAs as revealed by REPuter**. Repeated elements with identical sequences are connected on the circular representations of the genomes. Repeats larger than 30 bp and 45 bp are shown on the top and bottom panels, respectively. For these analyses, one copy of the IR sequence was deleted from the *Nephroselmis*, *Pseudendoclonium*, *Oltmannsiellopsis *and *Chlamydomonas *genomes.

SDRs have most probably played a major role in remodelling the chloroplast genome in UTC lineages. A correlation has been previously observed between the abundance of SDRs and the extent of gene rearrangements in UTC algal genomes [6]. This correlation still holds with the addition of *Oltmannsiellopsis *chloroplast genome sequence. The abundance of SDR elements in *Oltmannsiellopsis *cpDNA is comparable to that observed in *Pseudendoclonium *cpDNA (Figure 7) and genes have been rearranged to a similar extent in both genomes (Table 6). SDRs in green plant cpDNAs could serve as hot spots for nonhomologous recombinational events and lead to inversions and transpositions [15,30,31].

## Conclusion

Although the *Oltmannsiellopsis *chloroplast genome differs considerably from its *Pseudendoclonium *counterpart at the levels of intron content and gene order, the two ulvophyte genomes share similarities in gene content and quadripartite architecture. We conclude that the chloroplast genome of the last common ancestor of *Oltmannsiellopsis *and *Pseudendoclonium *contained a minimum of 108 genes, was loosely packed with coding sequences, carried only a few group I introns, and featured a quadripartite architecture that deviates from the ancestral type displayed by *Mesostigma *and *Nephroselmis *cpDNAs with regard to the transcription direction of the rRNA genes and the gene contents of the single copy regions. Given the phylogenetic positions of *Oltmannsiellopsis *and *Pseudendoclonium*, these genomic characters were undoubtedly present in the earliest-diverging members of the Ulvophyceae. Numerous changes were experienced by the chloroplast genome in the lineages leading to *Oltmannsiellopsis *and *Pseudendoclonium*; these include contraction/expansion of the IR, migration of genes from the ancestral LSC region toward the single copy region corresponding to the SSC, gene losses, intron gains/losses, and gene rearrangements within the IR and each of the single copy regions. Considering that the chloroplast genome of *Codium fragile *(Ulvales) is greatly reduced in size (only 89 kbp) and lacks an IR [27], many additional chloroplast gene losses and rearrangements probably occurred in some lineages of the Ulvophyceae.

Our comparative analysis of the *Oltmannsiellopsis *chloroplast genome with its chlorophyte counterparts strengthens the idea that the chloroplast genomes of early-diverging ulvophytes occupy an intermediate position between those of the trebouxiophyte *Chlorella *and the chlorophycean green alga *Chlamydomonas *with respect to the retention of ancestral features [6]. In the context of the debate on the branching order of UTC lineages [4-6], this analysis provides further support for the published phylogenetic analysis of mitochondrial gene sequences identifying the Trebouxiophyceae as a basal lineage relative to the Ulvophyceae and Chlorophyceae [5].

## Methods

### Isolation and sequencing of *Oltmannsiellopsis *cpDNA

*Oltmannsiellopsis viridis *was obtained from the National Institute for Environmental Studies of Japan (NIES 360) and grown in K medium [32] under 12 h light/dark cycles. Organellar DNA was isolated and sequenced as described previously [5]. Sequences were edited and assembled with SEQUENCHER 4.2.1 (GeneCodes, Ann Arbor, MI). The fully annotated chloroplast genome sequence has been deposited in [GenBank:DQ291132].

### Sequence analyses

Genes and ORFs were identified as described previously [6]. Homologous introns were detected by BLASTN searches [33] against the non-redundant database of National Center for Biotechnology Information using an *E *value threshold of 1 × 10^-4^. Homologous introns inserted at identical positions within the same gene were identified by manual screening of the GOBASE database [34].

Repeated sequences were mapped with PipMaker [35], identified with REPuter 2.74 [36] and classified with REPEATFINDER [37], using the default parameters. Sequences clustered with REPEATFINDER were aligned manually using BIOEDIT 7.0.1 [38], and non-overlapping SDR units were identified by manual screening of the alignment. Numbers of SDR units were determined with FINDPATTERNS of the GCG Wisconsin Package version 10.2 (Accelrys, Burlington, Mass.), using 100% or 90% sequence identity. Putative stem-loop structures and degenerate repeats were identified using PALINDROME and ETANDEM in EMBOSS 2.9.0 [39], respectively. The density of repeated elements in a given chloroplast genome was assessed with REPuter 2.74 [36] using the -f (forward), -p (palindromic), and -allmax options at minimum lengths (-l) of 30 bp and 45 bp. For the analyses involving IR-containing genomes, one copy of the IR sequence was deleted. Circle graphs generated by REPuter were screen-captured at 300 dpi and converted to black and white illustrations with GIMP 2.0 [40]. Repeated elements in different cpDNAs were compared using Vmatch [41] and GenAlyzer 0.81 b [42].

The GRIMM web server [43] was used to infer the minimal number of gene permutations by inversions in pairwise comparisons of chloroplast genomes. Because GRIMM cannot deal with duplicated genes and requires that the compared genomes have the same gene content, genes within one of the two copies of the IR were excluded and only the genes common to all the compared genomes were analysed. The data set used in the comparative analyses reported in Table 6 contained 90 genes; the three exons of the *trans*-spliced *psaA *gene were coded as distinct fragments (for a total of 92 gene loci).

## Abbreviations

cpDNA, chloroplast DNA; IR, inverted repeat; LSC, large single copy; ORF, open reading frame; rRNA, ribosomal rRNA; SDR, short dispersed repeat; SSC, small single copy; UTC, Ulvophyceae/Trebouxiophyceae/Chlorophyceae.

## Authors' contributions

JFP participated in the conception of this study, carried out the genome sequencing, performed all sequence analyses, annotated the genome, generated the figures, and drafted the manuscript. CL and MT conceived the study, contributed to the interpretation of the data, and helped to prepare the manuscript. All authors read and approved the final manuscript.

## Figures and Tables

**Table 1:** General features of *Oltmannsiellopsis *and other chlorophyte cpDNAs

**Feature**	***Nephroselmis***	***Chlorella***	***Oltmannsiellopsis***	***Pseudendoclonium***	***Chlamydomonas***
Size (bp)					
Total	200,799	150,613	151,933	195,867	203,827
IR	46,137	--^a^	18,510	6,039	22,211
LSC	92,126	--^a^	33,610	140,914	81,307^b^
SSC	16,399	--^a^	81,303	42,875	78,088^b^
A+T (%)	57.9	68.4	59.5	68.5	65.5
Coding sequences (%)^c^	68.7	60.9	59.2	62.3	50.1
Genes (no.)^d^	128	112	105	105	94
Introns (no.)					
Group I	0	3	5	27	5
Group II	0	0	0	0	2

^a ^Because *Chlorella *cpDNA lacks an IR, only the total size of this genome is given.

^b ^The LSC and SSC regions of *Chlamydomonas *cpDNA cannot be distinguished unambiguously.

^c ^Conserved genes, unique ORFs and introns were considered as coding sequences.

^d ^Genes present in the IR were counted only once. Unique ORFs and intron ORFs were not taken into account.

**Table 2:** Differences between the gene repertoires of *Oltmannsiellopsis *and other UTC algal cpDNAs

**Gene^a^**	***Chlorella***	***Oltmannsiellopsis***	***Pseudendoclonium***	***Chlamydomonas***
*accD*	●	●	●	○
*chlB*	●	●	○	●
*chlI*	●	●	●	○
*chlL*	●	●	○	●
*chlN*	●	●	○	●
*cysA*	●	○	○	○
*cysT*	●	○	○	○
*infA*	●	●	●	○
*minD*	●	●	●	○
*psaI*	●	●	●	○
*psaM*	●	●	●	○
*rpl12*	●	●	●	○
*rpl19*	●	●	●	○
*rpl32*	●	●	●	○
*ycf20*	●	●	●	○
*ycf62*	●	○	●	○
*trnL*(caa)	●	○	●	○
*trnL*(gag)	●	○	○	○
*trnR*(ccg)	●	○	●	○
*trnR*(ccu)	○	●	●	○
*trnS*(gga)	●	○	○	○
*trnT*(ggu)	●	○	○	○

^a ^A filled/open circle denotes the presence/absence of a gene. Only the genes that are missing in one or more genomes are indicated. A total of 91 genes are shared by all compared cpDNAs: *atpA*, *B*, *E*, *F*, *H*, *I*, *ccsA*, *cemA*, *clpP*, *ftsH*, *petA*, *B*, *D*, *G*, *L*, *psaA*, *B*, *C*, *J*, *psbA*, *B*, *C*, *D*, *E*, *F*, *H*, *I*, *J*, *K*, *L*, *M*, *N*, *T*, *Z*, *rbcL*, *rpl2*, *5*, *14*, *16*, *20*, *23*, *36*, *rpoA*, *B*, *C1*, *C2*, *rps2*, *3*, *4*, *7*, *8*, *9*, *11*, *12*, *14*, *18*, *19*, *rrf*, *rrl*, *rrs*, *tufA*, *ycf1*, *3*, *4*, *12*, *trnA*(ugc), *C*(gca), *D*(guc), *E*(uuc), *F*(gaa), *G*(gcc), *G*(ucc), *H*(gug), *I*(cau), *I*(gau), *K*(uuu), *L*(uaa), *L*(uag), *Me*(cau), *Mf*(cau), *N*(guu), *P*(ugg), *Q*(uug), *R*(acg), *R*(ucu), *S*(gcu), *S*(uga), *T*(ugu), *V*(uac), *W*(cca), *Y*(gua).

**Table 3:** Compared sizes of expanded genes in *Oltmannsiellopsis *and other UTC algal cpDNAs

**Gene**	***Chlorella***		***Oltmannsiellopsis***		***Pseudendoclonium***		***Chlamydomonas***	
	
	**Size (bp)**	**Expansion^a^**	**Size (bp)**	**Expansion^a^**	**Size (bp)**	**Expansion^a^**	**Size (bp)**	**Expansion^a^**
*cemA*	801	1.6	1059	2.2	909	1.8	1503	3.0
*ftsH*	5163	1.9	6879	2.6	7791	2.9	8916	3.3
*rpoA*	837	0.9	1527	1.6	1734	1.8	2213^b^	2.3
*rpoB*	3906	1.2	4251	1.3	6537	2.0	4967^c^	1.5
*rpoC1*	2511	1.3	3066	1.5	4737	2.4	5739^c^	2.9
*rpoC2*	4689	1.3	5580	1.5	10389	2.8	9363	2.5
*ycf1*	2460	2.0	2427	2.0	2505	2.0	5988	4.9

^a ^Size relative to the corresponding gene in *Mesostigma*. Each value was obtained by dividing the size of the UTC algal gene by the size of the *Mesostigma *gene.

^b ^The indicated size is derived from our unpublished sequence data. We found that a portion of the *rpoA *coding sequences is missing in [GenBank:NC_005353] as a result of a sequencing error introducing a frameshift mutation.

^c ^The indicated size includes the intergenic spacer separating the ORFs corresponding to the 5' and 3' portions of the gene.

**Table 4:** Group I introns in *Oltmannsiellopsis *cpDNA

			**ORF**		
			
**Designation**	**Subgroup^a^**	**Size (bp)**	**Location^b^**	**Conserved motif^c^**	**Size (codons)**
Ov.*petB*.1	IB	1322	L8	LAGLIDADG (2)	264
Ov.*psbA*.1	IA2	1127	L6	GIY-YIG	240
Ov.*rrl*.1	IB4	830	L8	LAGLIDADG (1)	167
Ov.*rrl*.2	IB4	1129	L9.1	LAGLIDADG (2)	250
Ov.*rrl*.3	IA3	767	L6	LAGLIDADG (1)	165

^a ^Introns were classified according to Michel and Westhof [44]. The subcategory of the Ov.*petB*.1 intron could not be identified unambiguously.

^b ^L followed by a number refers to the loop extending the base-paired region identified by the number.

^c ^The conserved motif in the predicted homing endonuclease is given, with the number of copies of the LAGLIDADG motif indicated in parentheses.

**Table 5:** Group I introns at identical gene locations in *Oltmannsiellopsis *cpDNA, other green algal cpDNAs and land plant cpDNAs

***Oltmannsiellopsis *intron**	**Homologous Intron**	
	
	**Green plant^a^/Intron number^b^**	**Accession number**
Ov.*psbA*.1	*Pseudendoclonium akinetum *i5 (U)	[GenBank:AY835431]
	*Chlamydomonas reinhardtii *i3 (C)	[GenBank:NC_005353]
		
Ov.*rrl*.1	*Chlamydomonas geitleri *(C)	[GenBank:L43353]
	*Dunaliella parva *i2 (C)	[GenBank:L43540]
	*Haematococcus lacustris *i3 (C)	[GenBank:L49151]
	*Chlamydomonas callosa *i3 (C)	[GenBank:L43501]
	*Chlamydomonas komma *i2 (C)	[GenBank:L43502]
	*Chlamydomonas mexicana *i1 (C)	[GenBank:L49148]
	*Chlamydomonas frankii *i2 (C)	[GenBank:L43352]
	*Chlamydomonas pallidostigmatica *i2 (C)	[GenBank:L43503]
	*Neochloris aquatica *i2 (C)	[GenBank:L49155]
	*Chlorosarcina brevispinosa *i2 (C)	[GenBank:L49150]
	*Pedinomonas tuberculata *i1 (?)	[GenBank:L43541]
	*Monomastix species *M722 i1 (P)	[GenBank:L44124]
	*Monomastix species *OKE-1 i1 (P)	[GenBank:L49154]
	*Pterosperma cristatum *(P)	[GenBank:L43359]
		
Ov.*rrl*.2	*Chlamydomonas monadina *i3 (C)	[GenBank:L49149]
	*Chlamydomonas humicola *(C)	[GenBank:L42989]
	*Dunaliella parva *i4 (C)	[GenBank:L43540]
	*Chlamydomonas zebra *(C)	[GenBank:L43356]
	*Chlamydomonas starrii *(C)	[GenBank:L43504]
	*Chlamydomonas frankii *i4 (C)	[GenBank:L43352|
	*Neochloris aquatica *i3 (C)	[GenBank:L49155]
	*Ankistrodesmus stipitatus *i2 (C)	[GenBank:L42984]
	*Stigeoclonium helveticum *i4 (C)	[GenBank:L49157]
	*Trebouxia aggregata *i2 (T)	[GenBank:L43542]
	*Trichosarcina mucosa *i1 (U)	[GenBank:AY008341]
	*Pedinomonas tuberculata *i3 (?)	[GenBank:L43541]
	*Monomastix species *OKE-1 i3 (P)	[GenBank:L49154]
		
Ov.*rrl*.3	*Chlamydomonas agloeformis *(C)	[GenBank:L43351]
	*Chlamydomonas callosa *i4 (C)	[GenBank:L43501]
	*Chlamydomonas iyengarii *(C)	[GenBank:L43354]
	*Chlamydomonas mexicana *i2 (C)	[GenBank:L49148]
	*Chlamydomonas nivalis *(C)	[GenBank:L42990]
	*Chlamydomonas peterfii *(C)	[GenBank:L43538]
	*Chlamydomonas reinhardtii *(C)	[GenBank:NC_005353]
	*Carteria lunzensis *(C)	[GenBank:L42986]
	*Carteria olivieri *(C)	[GenBank:L43500]
	*Haematococcus lacustris *i4 (C)	[GenBank:L49151]
	*Pediastrum biradiatum *(C)	[GenBank:L49156]
	*Neochloris aquatica *i4 (C)	[GenBank:L49155]
	*Scenedesmus obliquus *(C)	[GenBank:L43360]
	*Pseudendoclonium akinetum *(U)	[GenBank:AY835431]
	*Trichosarcina mucosa *i2 (U)	[GenBank:AY008341]
	*Chlorella vulgaris *(T)	[GenBank:NC_001865]
	*Monomastix species *M722 i3 (P)	[GenBank:L44124]
	*Monomastix species *OKE-1 i4 (P)	[GenBank:L49154]
	*Scherffelia dubia *(P)	[GenBank:L44126]
	*Anthoceros punctatus *(E)	[GenBank:AF393576]
	*Anthoceros formosae *(E)	[GenBank:AB086179]

^a ^The letter in parentheses indicates the specific chlorophyte/streptophyte lineage comprising the green algal/plant indicated. P, Prasinophyceae; U, Ulvophyceae; T, Trebouxiophyceae; C, Chlorophyceae; E, Embryophyta; and ?, uncertain affiliation within the Chlorophyta.

^b ^The intron number is given when more than one intron is present.

**Table 6:** Minimal numbers of inversions accounting for gene rearrangements between green algal cpDNAs

**Compared cpDNA**	**Number of inversions^a^**				
	
	***Mesostigma***	***Nephroselmis***	***Chlorella***	***Oltmannsiellopsis***	***Pseudendoclonium***
*Chlamydomonas*	75	74	75	75	77
*Pseudendoclonium*	54	55	52	55	
*Oltmannsiellopsis*	54	55	50		
*Chlorella*	46	47			
*Nephroselmis*	43				

^a ^Numbers of gene permutations by inversions were computed using GRIMM.

**Table 7:** SDR units in *Oltmannsiellopsis *cpDNA

			**Number^a^**	
			
**Designation**	**Size (bp)**	**Sequence**	**100%**	**90%**
A	11	CAACACTYCCA	252	426
B	11	AWAGCGAAGCW	89	126
C	7	GCCCCCC	126	126
D	8	GGGGAGGG	45	45
E	21	AGGGGCTTTGCTTCGCGGTTT	17	17

^a ^Numbers of SDR units were determined in searches performed using 100% or 90% sequence identity. The genomic locations of these repeated elements are reported in [GenBank: DQ291132].
